# Supplementary material for: Substance use among young people in the West Arsi Zone, Ethiopia: A cross-sectional study
Source: PLoS One. 2025 Mar 28;20(3):e0319432. doi: 10.1371/journal.pone.0319432 (PMC11952231; doi:10.1371/journal.pone.0319432)
Supplement: S1 Table — Proportional distribution of sample size among the chosen kebeles, West Arsi zone, Ethiopia, 2023 (N = 427). (DOCX) [file pone.0319432.s001.docx]

S1 Table: Proportional distribution of sample size among the chosen kebeles, West Arsi zone, Ethiopia, 2023 (N=427)

| S.# | Selected Woreda | Woreda total population | Selected Kebele | Sample size from each kebele | Total sample from each woreda |
| --- | --- | --- | --- | --- | --- |
| 1 | Shashemene | 158,357 | Awasho (*U*) | 43 | 143 (*141 included in the analysis*) |
|  |  |  | Dida boke (*U*) | 38 |  |
|  |  |  | Faji goba (*R*) | 33 |  |
|  |  |  | Borera (*R*) | 29 |  |
| 2 | Adaba | 94,451 | Wosha (*R*) | 22 | 88 |
|  |  |  | Bucha raya (*R*) | 21 |  |
|  |  |  | Kebele 01 (*U*) | 23 |  |
|  |  |  | Kebele 02 (*U*) | 22 |  |
| 3 | Wondo | 87,484 | Gotu (*R*) | 17 | 79 |
|  |  |  | Shasha (*R*) | 18 |  |
|  |  |  | Hintaye (*U*) | 21 |  |
|  |  |  | Busa (*U*) | 23 |  |
| 4 | Negelle Arsi | 129,565 | Kersa ilala (*R*) | 24 | 117 (*116 included in the analysis*) |
|  |  |  | Melka shehiti (*U*) | 31 |  |
|  |  |  | Kiltu dema (*U*) | 32 |  |
|  |  |  | Ali woyo (*R*) | 30 |  |
|  | *4 woredas* |  | *16 kebeles* | *427* | *427* |

R= Rural U= Urban
